# Molecular characterization of IncFII plasmid carrying *bla*_NDM-5_ in a *Salmonella enterica* serovar Typhimurium ST34 clinical isolate in China

**DOI:** 10.1128/msphere.00480-23

**Published:** 2023-11-01

**Authors:** Shihan Zeng, Yulan Huang, Xiwei Zhang, Liang Fu, Zhaohui Sun, Xiaoyan Li

**Affiliations:** 1 Department of Clinical Laboratory, Fifth Affiliated Hospital, Southern Medical University, Guangzhou, China; 2 Department of Laboratory Medicine, General Hospital of Southern Theater Command, Guangzhou, China; JMI Laboratories, North Liberty, Iowa, USA

**Keywords:** *bla*_NDM-5_, *S. enterica *serovar Typhimurium, IncFII plasmid, carbapenem-resistance, IS*26*

## Abstract

The detection rate of carbapenem-resistant nontyphoidal *Salmonella* (NTS) is relatively low. However, carbapenem-sensitive and carbapenem-resistant *Salmonella enterica* serovar Typhimurium (*S. enterica* serovar Typhimurium) were isolated from a clinical outpatient within a span of 10 days, named 1104–65 and 1104–75. We aimed to reveal the mechanism of carbapenem resistance in *S. enterica* serovar Typhimurium isolates in this case. The resistance phenotype of *S. enterica* serovar Typhimurium was analyzed by the VITEK-2 Compact equipment and Kirby-Bauer disc diffusion method. Whole-genome sequencing was used to analyze the serotype, ST type, genetic relationship, resistance genes, plasmid replicon, the genetic environment of *bla*_NDM-5_, and the characteristics of IncFII plasmid carrying *bla*_NDM-5_ in *S. enterica* serovar Typhimurium. The transferability of the IncFII plasmid carrying *bla*_NDM-5_ was verified by the conjugation assay and PCR. The serotypes of both NTS are *S. enterica* serovar Typhimurium, belonging to ST34. Single nucleotide polymorphisms analysis showed that they were the same clone. A conjugative IncFII plasmid pIncFII-NDM5 with transferability was detected in isolate 1104–75, which harboring the *bla*_NDM-5_ gene was the primary mechanism responsible for mediating carbapenem resistance in *S. enterica* serovar Typhimurium. The genetic environment of *bla*_NDM-5_ on pIncFII-NDM5 is “IS*26*-ΔIS*3000*-IS*5*-ΔIS*Aba125-bla*_NDM-5_-*ble*_MBL_-*trpF-dsbC -cutA*-IS*26*”, which was confirmed as a novel structure not previously reported in the NCBI database. Although the mechanism is rarely reported in NTS, the prevalence of IncFII plasmid pIncFII-NDM5 will pose a great threat to the clinical treatment of *S. enterica* serovar Typhimurium. Meanwhile, the coexistence of *bla*_CTX-M-55_, *qnrS1*, *bla*_NDM-5_, and *mph(A*) in *S. enterica* serovar Typhimurium ST34 warrants additional attention.

**IMPORTANCE:**

In this study, an IncFII plasmid pIncFII-NDM5 carrying *bla*_NDM-5_ was found in carbapenem-resistant *Salmonella enterica* serovar Typhimurium (*S. enterica* serovar Typhimurium), which has conjugative transferability and carried *bla*_NDM-5_, *ble*_MBL_, *mph(A*), and *bla*_TEM-1_ four resistance genes that can mediate resistance to multiple antibiotics including cephalosporins, beta-lactamase inhibitor combinations, carbapenems, and macrolides. Phylogenetic analysis showed that 1104–65 and 1104–75 were closely related to other *S. enterica* serovar Typhimurium in this area. The above-mentioned *S. enterica* serovar Typhimurium chromosome carries *bla*_CTX-M-55_, *qnrS1*, and *tet(A*) genes, so the antibiotic resistance of isolates will be further enhanced after obtaining the pIncFII_NDM5-like plasmid. Meanwhile, we discovered a novel genetic structure of *bla*_NDM-5_ mediated by the IS*26* composite transposon, which will expand our understanding of the emergence and spread of carbapenem-resistance genes. Altogether, the presence of the IncFII plasmid pIncFII-NDM5 further underscores the need for vigilant surveillance and appropriate infection control measures to mitigate the impact of carbapenem-resistant *S. enterica* serovar Typhimurium in clinical settings.

## INTRODUCTION

Nontyphoidal *Salmonella* (NTS) is a prevalent bacterial cause of gastrointestinal diseases worldwide, with over 2,600 serotypes. *Salmonella* Typhimurium (*Salmonella enterica* serovar Typhimurium) is one of the most common serotypes, infecting both humans and animals (1, 2). NTS infection is a self-limiting disease, but in immunocompromised persons, such as children and the elderly, it can progress to severe systemic disease and antibiotic therapy is also necessary (3, 4). During recent decades, as the primary antibiotic therapy choices for NTS, fluoroquinolones, and extended-spectrum cephalosporins (ESCs) were used in anti-infective therapy more frequently, which has led to an increasing resistance rate in NTS (5–7). Carbapenem antibiotics are β-lactam antibiotics with broad activity, which are specially used for the treatment of severe bacterial infections (8, 9). Therefore, carbapenem antibiotics may be the last resort for patients with aggressive, multidrug-resistant NTS infections (10).

Although carbapenem-resistant NTS is still extremely rare, it will pose a serious threat to antimicrobial therapy once it occurs (11, 12). Several carbapenemases have been reported in NTS, including KPC, IMP, NDM, VIM, and OXA-48 (11, 13–15). Here, we report an NDM-5-producing carbapenem-resistant *S. enterica* serovar Typhimurium from an outpatient. With two amino acid changes (Val88Leu and Met154Leu), the NDM-5 variant of NDM-1 has a higher resistance to carbapenems and ESCs than NDM-1 (16). Since it was first discovered in *Escherichia coli* (*E. coli*) in 2011, NDM-5 carbapenemase has occasionally been discovered in other *Enterobacteriaceae*, such as *Klebsiella pneumoniae* (*KP*) and NTS (11). In this case, a carbapenem-resistant clinical *S. enterica* serovar Typhimurium was discovered in the Fifth Affiliated Hospital, Southern Medical University located in Conghua District, Guangzhou in November 2021. Therefore, this study aims to investigate the mechanism of carbapenem resistance in a clinical *S. enterica* serovar Typhimurium isolate.

## RESULTS

### Isolate identification and antimicrobial susceptibility testing results

Two NTS strains were successively isolated from the stool samples of the same outpatient. In November 2021, strain 1104–65 was isolated from the patient’s stool, followed by strain 1104–75 in another stool specimen 10 days later. Unfortunately, we did not collect further details about this outpatient treatment, so it is unclear what the patient’s treatment program was during 10 days. Both isolates were typed *S. enterica* serovar Typhimurium (O4: Hi). The antibiotic susceptibility results are shown in Table 1. Compared with 1104–65, isolate 1104–75 had increased minimum inhibitory concentration (MIC) to ceftazidime (CAZ), cefepime (FEP), and azithromycin (AZM), among which FEP became resistant (MIC ≥32 µg/mL), and was resistant to cefoxitin (FOX), amoxicillin-clavulanic acid (AMC), piperacillin-tazobactam (TZP), imipenem (IPM), and ertapenem (ETP). Both isolates were intermediary to levofloxacin (LVX) and ciprofloxacin (CIP). The resistance profiles of 1104–65 and 1104–75 to other antibiotics were broadly similar.

**TABLE 1:** The antibiotic susceptibility results of the isolates^*a*^

Isolate	MIC (µg/mL)												Zone diameter (mm)			
	CXM	CRO	CAZ	FEP	FOX	AMC	TZP	LVX	SXT	IPM	ETP	TGC	AMP	CIP	AZM	CHL
1104–65	≥64	≥64	32	8	≤4	4	≤4	1	≤1	≤0.25	≤0.12	1	6	27	11	26
1104–75	≥64	≥64	≥64	≥32	≥64	≥32	128	1	≤1	≥16	≥8	≤0.5	6	28	6	21
C600	16	≤0.25	0.5	≤0.12	8	4	≤4	0.5	≤1	≤0.25	≤0.12	≤0.5	14	29	25	24
C-1104–75	≥64	≥64	≥64	16	≥64	≥32	≥128	0.5	≤1	≥16	≥8	≤0.5	6	28	8	24

^*a*^ CXM, cefuroxime; CRO, ceftriaxone; SXT, cotrimoxazole; TGC, tigecycline; AMP, ampicillin; CHL, chloramphenicol.

### Plasmid and resistance gene analysis

The whole genome sequence revealed that compared with isolate 1104–65, isolate 1104–75 additionally harbored IncFII plasmid replicon and Col156_1. In addition, 1104–75 also possessed four resistant genes (*bla*_NDM-5_, *ble*_MBL_, *mph(A*), and *bla*_TEM-1_, which, respectively, confer resistance to carbapenems, bleomycin, and macrolides), all of which were located on the IncFII plasmid (named pIncFII-NDM5). Both chromosomes carried *tet(B*), *bla*_CTX-M-55_, and *qnrS1* resistance genes.

### Phylogenetic analysis

The bioinformatics analysis showed that the serotype 1104–65 and 1104–75 both were *S. enterica* serovar Typhimurium, belonging to ST34. The phylogenetic relationship of 1104–65, 1104–75, and other *S. enterica* serovar Typhimurium (*n* = 67) in this area from our previous study were assessed using *S. enterica* serovar Typhimurium ATCC14028 as a reference strain (see Fig. S1). Sixty-seven strains of other *S. enterica* serovar Typhimurium in this area were isolated from the stools of patients from May 2020 to February 2021 in the Fifth Affiliated Hospital, Southern Medical University (for details, see Table S1). Single nucleotide polymorphisms (SNPs) analysis showed that there were 17 SNPs between 1104-65 and 1104–75, indicating that they belonged to the same clone. Meanwhile, the SNPs between the two and other *S. enterica* serovar Typhimurium ST34 in this area ranged from 0 to 157, showing a close genetic relationship. It is worth noting that the resistance gene spectrum of 1104–65 is consistent with S24, S79, S36, S49, S34, S42, and S133. All of these strains carry *bla*_CTX-M-55_, *qnrS1*, and *tet(B*). Moreover, our previous studies have shown that these three resistance genes were located on the chromosome of *S. enterica* serovar Typhimurium and can be transmitted vertically.

### Characterization of plasmid pIncFII-NDM5

The transconjugant was successfully obtained through the conjugation experiment and named C-1104–75. PCR and sequencing results showed that the transconjugant carried IncFII plasmid replicon and *bla*_NDM-5_ gene, which indicates that the plasmid pIncFII-NDM5 carrying the *bla*_NDM-5_ gene is a conjugative plasmid with transferability. The drug susceptibility results of C-1104–75 are shown in Table 1. Compared with the recipient strain *E. coli* C600, pIncFII-NDM5 confers the transconjugant with resistance to ESCs, FOX, AMP, AMC, TZP, IPM, ETP, and AZM.

**Fig 1:**
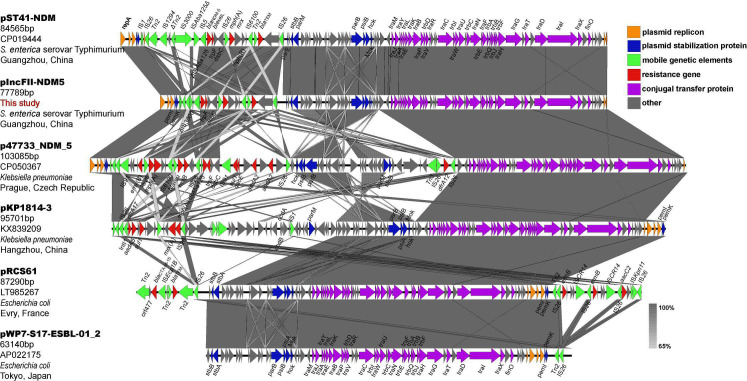
Linear comparison of plasmid pIncFII-NDM5 with other similar IncFII plasmids (with or without *bla*_NDM-5_). Gray shading indicates regions of shared homology among different elements. Open reading frames are marked by colored arrows, orange indicates genes encoding plasmid replicon, blue indicates genes encoding plasmid stabilizing proteins, green indicates mobile genetic elements, red indicates resistance genes, purple indicates genes encoding conjugal transfer proteins, and gray indicates other genes.

The plasmid pIncFII-NDM5 has a total length of 77,785 bp. Download the sequence of IncFII plasmids (with or without *bla*_NDM-5_) similar to pIncFII-NDM5 in other studies through NCBI: (I) Plasmid pST41-NDM (no. CP016389) was isolated from *S. enterica* serovar Typhimurium detected in stool samples of children with acute diarrhea in Guangzhou, China. The full length is 84,565 bp, carrying four resistance genes *bla*_NDM-5_, *ble*_MBL_, *mph(A*), and *bla*_TEM-1_. (II) Plasmid p47733_NDM_5 (no.CP050367) was isolated from *KP* and detected in rectal swab of patients in a hospital in Prague, Czech Republic. The full length is 103,085 bp, carrying nine resistance genes *erm(B*), *mph(A*), *bla*_TEM-1_, *rmt(B*), *bla*_NDM-5_, *ble*_MBL_, *sul1*, *aadA2* (2 copy number), and *dfrA12*. (III) Plasmid pKP1814-3 (no. KX839209) was isolated from *KP* detected in a hospital in Hangzhou, China. The full length is 95,701 bp, carrying five resistance genes *dfrA17*, *aadA2*, *sul1*, *erm(B*), and *mph(A*). (IV) Plasmid pRCS61 (no. LT985267) was isolated from *E. coli* in Evry, France. The full length is 87,290 bp, carrying four resistance genes *bla*_CTX-M-15_, *bla*_TEM_, *erm(B)* (2 copy number), and *aacC2*. (V) Plasmid pWP7-S17-ESBL-01_2 (no. AP022175) was isolated from *E. coli* detected in waste water treatment plant effluent in Tokyo, Japan. The full length is 63,140 bp, with no resistance genes present. Through the comparison of pIncFII-NDM5 and the above IncFII plasmid sequence (Fig. 1), it was found that the backbone structure of IncFII plasmids was almost identical, mainly including related genes encoding proteins involved in replication, maintenance and conjugative transfer. However, most of the mobile genetic elements and resistance genes were located in the variable region of IncFII plasmids. It can be seen from Fig. 1 that the two IncFII plasmids carrying *bla*_NDM-5_ from *S. enterica* serovar Typhimurium are highly similar in variable regions, but the mobile genetic elements of pST41-NDM are more abundant than pIncFII-NDM5. Notably, the IS*3000* upstream of *bla*_NDM-5_ of pIncFII-NDM5 was incomplete (469/3235), and one end of the IS*Aba125* sequence truncated by IS*5* was also missing. It indicates that the IncFII plasmid carrying *bla*_NDM-5_ is still evolving in *S. enterica* serovar Typhimurium. Simultaneously, the gene encoding the conjugative transfer-associated protein of IncFII plasmid confers it with conjugative transferability, which will pose a significant clinical risk. In addition, IS*26* appears to play an important role in the acquiring of resistant gene segments in the variable region.

### Comparative analysis of the genetic environment of *bla*_NDM-5_

**Fig 2:**
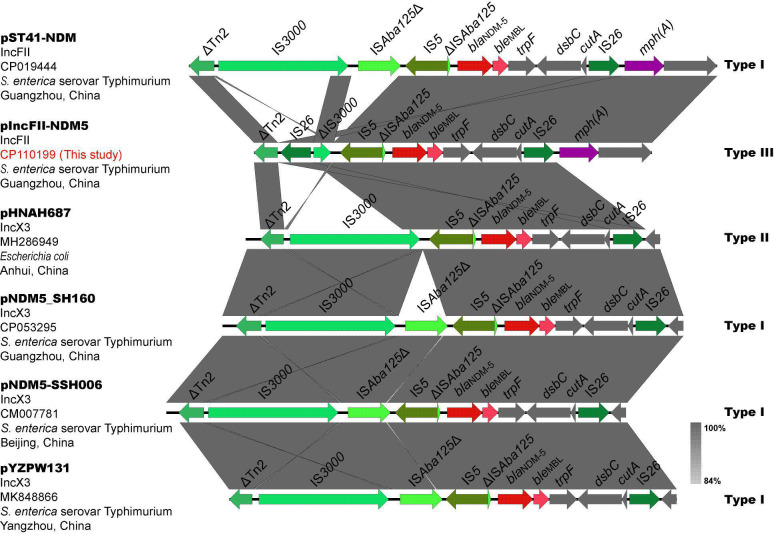
The genetic environments of *bla*_NDM-5._ Different colored arrows represent various open reading frames (ORFs), and the arrow’s direction indicates the direction of transcription. Homogeneous regions are represented by light gray shadows.

The genetic environment of *bla*_NDM-5_on pIncFII-NDM5 was intercepted for correlation analysis, about 9256 bp, compared with the BLAST database (http://www.ncbi.nlm.nhi.gov/blast/) and downloaded the plasmid sequence (GenBank accession number MH286949) of the most similar fragment. Meanwhile, literatures of *S. enterica* serovar Typhimurium carrying *bla*_NDM-5_ were searched through the PubMed database (https://www.ncbi.nlm.nih.gov/pubmed/), and the plasmid sequences carrying *bla*_NDM-5_ were downloaded to compare and analyze the genetic environment of *bla*_NDM-5_ (see Fig. 2). The relevant information of the above plasmids is shown in Table 2. Three different genetic environments were found surrounding *bla*_NDM-5_.

**TABLE 2:** Information about plasmid carrying *bla*_NDM-5_ in Fig. 2

Name	Isolate	Inc^*a*^	Origin	Country	Year of isolation	Reference	Types of *bla*_NDM-5_ genetic structure^*c*^
pNDM5-SSH006	*S. enterica* serovar Typhimurium	IncX	Patient feces	Shanghai, China	2015	(17)	Ⅰ
pNDM5_SH160	*S. enterica* serovar Typhimurium	IncX	Retail pork	Shanghai, China	2016	(18)	Ⅰ
pST41-NDM	*S. enterica* serovar Typhimurium	IncFII	Patient feces	Guangzhou, China	2017	(13)	Ⅰ
pHNAH687	*E. coli*	Inc X	Chicken feces	Anhui, China	2018	BLAST^*b*^	Ⅱ
pYZPW131	*S. enterica* serovar Typhimurium	IncX	Retail pork	Jiangsu, China	2019	(14)	Ⅰ
pIncFII-NDM5	*S. enterica* serovar Typhimurium	IncFII	Patient feces	Guangzhou, China	2021	This study	Ⅲ

^*a*^ Inc, incompatibility group.

^*b*^ BLAST, the plasmid carrying the region most similar to the *bla*_NDM-5_ gene environment on pIncFII-NDM5 by BLAST search on the NCBI database.

^*c*^ Type Ⅰ was “IS*3000*-IS*Aba125*Δ-IS*5*-ΔIS*Aba125*-*bla*_NDM-5_-*ble*_MBL_-*trpF*-*dsbC*-*cutA*-IS*26*”, type Ⅱ was “IS*3000*-IS*5*-ΔIS*Aba125*-*bla*_NDM-5_-*ble*_MBL_-*trpF* -*dsbC*-*cutA*-IS*26*”, and type Ⅲ was “IS*26*-ΔIS*3000*-IS*5*-ΔIS*Aba125*-*bla*_NDM-5_-*ble*_MBL_-*trpF*-*dsbC*-*cutA*-IS*26*”.

Type I “IS*3000*-IS*Aba125*Δ-IS*5*-ΔIS*Aba125-bla*_NDM-5_-*ble*_MBL_-*trpF-dsbC-cutA*- IS*26*” was the most common type of structure, discovered in the IncFII plasmid pST41-NDM and three IncX3 plasmids isolated from *S. enterica* serovar Typhimurium. The genetic structure of *bla*_NDM-5_ on the IncFII plasmid pIncFII-NDM5 isolated in this study was type III “IS*26*-ΔIS*3000*-IS*5*-ΔIS*Aba125-bla*_NDM-5_-*ble*_MBL_-*trpF-dsbC-cutA*-IS*26*”, while the most similar structure on the IncX3 plasmid pHNAH687 isolated from *E. coli* was type II “IS*3000*-IS*5*-ΔIS*Aba125-bla*_NDM-5_-*ble*_MBL_-*trpF-dsbC-cutA*-IS*26*”. The differences between the three types were IS*3000* (complete/incomplete), IS*26* (presence/absence), and one end of IS*Aba125* truncated by IS*5* (presence/absence). Beyond comprehensive analysis, we speculate that type I evolved into type II after the loss of IS*Aba125*Δ (1–1018/1087). After a new IS*26* was inserted and truncated into IS*3000* of type II, it formed a composite transposon with IS*26* downstream of *bla*_NDM-5_ to mediate the transfer of *bla*_NDM-5_, and type II evolved into type III. To our knowledge, the IS*26* composite transposon has never been described to mediate *bla*_NDM-5_ transfer in previous studies.

## DISCUSSION

NTS is a major cause of foodborne illness in animals and humans worldwide. With the emergence and rapid development of NTS-resistance phenotype ACSSuT (defined as resistance to AMP, CHL, streptomycin, sulfamethoxazole, and tetracycline), fluoroquinolones (FQs) and ESCs are commonly used as a first-line agent for the treatment of NTS infections (17–21). However, with widespread use, the detection rate of NTS resistant to ESCs and QRs has been increasing in recent years (1, 22–25). Therefore, carbapenems may be the last resort for patients with invasive, multidrug-resistant (MDR, resistance to three or more classes of antimicrobials) NTS infection (26).

Resistance to carbapenems in *Enterobacteriaceae* occurs involves multiple mechanisms, such as production of carbapenemases, production of extended-spectrum β-lactamases (ESBLs) or AmpC enzymes combined with the loss of specific outer membrane porins, increased efflux pump activity (12, 27, 28). The drug resistance mechanisms mentioned above are commonly observed in *E. coli* and *KP* but are rarely reported in NTS (11, 29, 30). The first carbapenemase gene isolated in NTS was *bla*_KPC-2_, which was found in *S. enterica* serovar Cubana isolated from the stool of a 4-year-old boy with diarrhea in the United States in 1998 (31). Subsequently, carbapenemase genes *bla*_IMP-4_, *bla*_NDM-1_, *bla*_NDM-5_, *bla*_VIM-2_, and *bla*_OXA-48_ were successively reported in NTS (11). Carbapenem-resistant NTS has become a serious clinical problem due to limited treatment options. In this study, the mechanism that mediates the resistance of *S. enterica* serovar Typhimurium clinical isolate 1104–75 to carbapenems is the production of NDM-5 carbapenemase.

NDM-5 carbapenemase is currently primarily detected in *E. coli* and is still uncommon in other *Enterobacteriaceae* such as NTS and *KP* (32). The most prevalent plasmid type in *Enterobacteriaceae* to contain *bla*_NDM-5_ is IncX3 (33, 34). Compared with 1104–65, the MIC of 1104–75 carrying *bla*_NDM-5_ to ESCs, β-lactam/β-lactamase inhibitor, and carbapenems increased significantly, which was consistent with previous research results (13, 16, 35). The *bla*_NDM-5_ in this study is located on the IncFII plasmid pIncFII-NDM5 of *S. enterica* serovar Typhimurium clinical isolate 1104–75. By searching the PubMed database, it is found that *bla*_NDM-5_ is also mainly located on the IncX3 plasmid in *Salmonella* (14, 36–39). Only one article (13) reported that *bla*_NDM-5_ was localized on the IncFII plasmid pST41-NDM in *S. enterica* serovar Typhimurium isolated from a stool of a child with acute diarrhea in Guangzhou, China. The IncFII plasmid has a narrow host range was commonly found in *E. coli*, and has been involved in the global spread of the *bla*_CTX-M-15_ gene in the *E. coli* clone ST131 (40). Notably, pIncFII-NDM5 carrying the *bla*_NDM-5_ gene is a transferable conjugative plasmid that confers high levels of resistance to ESCs and carbapenems in clinical *S. enterica* serovar Typhimurium isolate 1104–75. Through the comparison of plasmid sequences, it was found that the backbone of pIncFII-NDM5 was very similar to other IncFII plasmids, with most genes encoding conjugative transfer proteins, which may be the main reason for the conjugative transferability of pIncFII-NDM5 (41). The IncFII plasmid pIncFII-NDM5 and pST41-NDM also carry the *mph(A*) gene, which confers AZM resistance. AZM is FDA-approved for the treatment of systemic *Salmonella* infections, particularly those caused by *S. enterica* serovar Typhimurium, due to increased rates of resistance to ESCs and FQs (42). Meanwhile, AZM is widely used in the treatment of various infections in children due to it is well tolerated in the presence of multiple co-morbidities and medications (43, 44). Additionally, it is worth noting that the genetic structure “IS*5*-ΔIS*Aba125-bla*_NDM-5_-*ble*_MBL_-*trpF-dsbC-cutA* -IS*26 -mph(A)-mrx-mph(R*)-IS*6100*” located on the pIncFII-NDM5 and pST41-NDM plasmids are completely identical, about 9096 bp. Through the BLAST tool, it was found that this framework also exists on the IncFII plasmid pGZ_NDM5 (no. CP017981) in *E. coli*. Although the *bla*_NDM-5_ and *mph(A*) gene combinations have been reported in previous literature, it has not been noticed both are located on the same genetic framework (45, 46). This suggests that clinicians should be alert to the phenomenon of co-transfer of *bla*_NDM-5_ and *mph(A*).

*S. enterica* serovar Typhimurium has a broad host range and is one of the major NTS serotypes responsible for outbreaks of infectious diarrhea and foodborne disease worldwide (47). ST34 is the most common ST type of *S. enterica* serovar Typhimurium and is often associated with ACSSuT resistance patterns (48). In this study, 1104–75 and 1104–65 have a relatively close genetic relationship with other *S. enterica* serovar Typhimurium ST34 isolates in this area (see Fig. S1). This means that pIncFII-NDM5 will be extremely dangerous if it becomes widespread among *S. enterica* serovar Typhimurium ST34 in this area. In our previous studies, it has been reported that there may be a potential epidemic clone of *S. enterica* serovar Typhimurium ST34 in this region with *bla*_CTX-M-55_ and *qnrS1* localized on the chromosome (49). The *qnrS1* gene can mediate low-level **r**esistance to FQ, and its presence can provide a selective advantage for strains exposed to FQs, thereby accelerating the development of chromosome-mediated FQs resistance in strains (50). More importantly, this study reported the emergence of NTS carrying four resistance genes (*bla*_CTX-M-55_, *qnrS1*, *bla*_NDM-5_, and *mph(A*)). Thus, the phenomenon of 1104–65-like clone *S. enterica* serovar Typhimurium ST34 carrying pIncFII_NDM5-like plasmid warrants additional attention because it may accelerate the development and spread of NTS coresistant to ESCs, FQs, carbapenems, and macrolide antibiotics.

The most typical *bla*_NDM-5_ genetic structure, Type I “IS*3000*-IS*Aba125*Δ-IS*5*-ΔIS*Aba125-bla*_NDM-5_-*ble*_MBL_-*trpF-dsbC-cutA*-IS*26*”, is frequently observed in IncX3 plasmids (51, 52). Of note, the genetic environment (type I) of *bla*_NDM-5_ on the IncFII plasmid pST41-NDM was the same as the genetic environment of *bla*_NDM-5_ on the IncX3 plasmid carried by other Enterobacteriaceae (*E. coli*, *KP,* and *Enterobacter cloacae*) from the same hospital. It suggested that the genetic environment of *bla*_NDM-5_ in the variable region of pST41-NDM may be derived from the IncX3 plasmid (13). The genetic structure of *bla*_NDM-5_ on the IncFII plasmid pIncFII-NDM5 isolated in this study was type III “IS*26*-ΔIS*3000*-IS*5*-ΔIS*Aba125-bla*_NDM-5_-*ble*_MBL_-*trpF-dsbC-cutA*-IS*26*”. Compared with another IncFII plasmid pST41-NDM, pIncFII-NDM5 has a new IS*26* inserted and truncated IS*3000* (2754–3222/3235), and one end of IS*Aba125* (1–1018/1087) was missing in the genetic structure. From type I to type III, we speculate that there are two main steps. Meanwhile, it was brought to our attention that IS*3000*on pST41-NDM, pNDM5_SH160, pNDM5-SSH006, and pYZPW131 was complete, whereas IS*3000*on pHNAH687 was incomplete (1–3222/3235), which provides stronger evidence for our conjecture. Mediated by mobile elements, the genetic environment of *bla*_NDM-5_ is constantly changing during the transfer process. Currently, the genetic structure of *bla*_NDM-5_ shares two common features (33, 39): (I) the insertion sequence IS*Aba125* (complete or truncated) is present upstream of *bla*_NDM-5_. (II) The downstream of *bla*_NDM-5_ includes the *ble*_MBL_ gene that mediates bleomycin resistance, followed by *trpF* (encoding phosphoribosyl anthranilate isomerase), *dsbC* (also known as *tat*, encoding a twin-arginine translocation pathway signal sequence domain protein), and *cutA* (also known as *dct*, encoding a periplasmic divalent cation tolerance protein). According to reports, *trpF* and *dsbC* play key roles in the stability, retention, or spread of *bla*_NDM-5_, or promotion of enzyme function (53). Currently, insertion sequences found in the genetic environment of *bla*_NDM-5_ include IS*Aba125* (complete or truncated), IS*91*, IS*26*, IS*5*, IS*3000* (complete or truncated), and IS*CR1* (54–60). IS*26*, a member of the IS*6* insertion sequence family, promotes the spread of antibiotic-resistance genes in Gram-negative bacteria mainly through the formation of composite transposons (61). Two IS*26*s in the same or opposite direction often form a composite transposon to mediate the transfer of resistance genes between them (62). For example, the IS*26* composite transposon in this study is involved in the mobilization of *bla*_NDM-5_. Its flanking elements are frequently deleted when IS*26* is inserted (63). This may be the reason why IS*3000* (2754–3222/3235) was truncated in Type III. A higher copy number of IS*26* was found in the variable region of IncFII plasmids in Fig. 1, which may be involved in the recombination of plasmids MDR region, thereby endowing isolates with resistance to multiple antibacterial drugs, ultimately limiting clinical treatment options. All in all, various evidences indicated that the genetic environment of *bla*_NDM-5_ composed of the IS*26* composite transposon is identified clinically for the first time in this study.

### Conclusion

In this study, an IncFII plasmid pIncFII-NDM5 carrying *bla*_NDM-5_ was isolated from *S. enterica* serovar Typhimurium detected from a stool sample of an outpatient in Conghua District, Guangzhou, which mediated resistance to carbapenems in *S. enterica* serovar Typhimurium. The genetic environment of *bla*_NDM-5_ “IS*26*-ΔIS*3000*-IS*5*-ΔIS*Aba125-bla*_NDM-5_-*ble*_MBL_-*trpF-dsbC-cutA*-IS*26*” was different from the previous typical structure, and IS*26* at both ends constitutes a composite transposon to mediate the gene transfer, which is also the first report of this type of genetic environment in *bla*_NDM-5_. Currently, reports of IncFII plasmids carrying *bla*_NDM-5_ in NTS are still rare. Our results suggest that the IncFII plasmid carrying *bla*_NDM-5_ may still be evolving and this type of plasmid can mediate high levels of resistance to ESCs and carbapenem. Meanwhile, *bla*_CTX-M-55_, *qnrS1*, *bla*_NDM-5_, and *mph(A*) cotransfer warrants additional attention because it may accelerate the development and spread of NTS coresistant to ESCs, FQs, carbapenems, and macrolide antibiotics.

## MATERIALS AND METHODS

### Bacterial collection, culture, and identification

The carbapenem-sensitive isolate 1104–65 and carbapenem-resistant isolate 1104–75 used in this study were collected from the stool of the same outpatient in Fifth Affiliated Hospital, Southern Medical University in Conghua District, Guangzhou. Strain 1104–65 was isolated from the patient’s stool in November 2021, and 1104–75 was isolated from another stool specimen 10 days later. Extract a sufficient amount of stool sample and use an inoculation loop to inoculate it onto blood agar plates, SS medium, and MacConkey agar plates. The typical colony morphology of *Salmonella* on SS medium is colorless, transparent, and black in the center. After incubation at 37°C for 16–18 hours, a single colony was selected and drawn on a blood agar plate to obtain pure isolates for identification and antimicrobial susceptibility tests. Isolates were analyzed and identified by the VITEK-2 COMPACT automatic microbial identification system (bioMérieux, Marcy-l'Étoile, France). *Salmonella* serotyping was conducted by using the slide agglutination test with specific antisera (Tianrun, Ningbo, China) according to the manufacturer’s instructions.

### Antimicrobial susceptibility testing

The MIC values for CTX, CRO, CAZ, FEP, FOX, AMC, TZP, LVX, SXT, IPM, ETP, and TGC were performed using the VITEK-2 Compact equipment. The diameter of the inhibition zone (mm) of the NTS isolates against AMP, CIP, AZM, and CHL was determined by the Kirby-Bauer disc diffusion method on Muller–Hinton (MH) agar plates. All of the procedures and results interpretation were followed by the Clinical and Laboratory Standards Institute (CLSI M100, 33th edition) guidelines.

### Whole-genome sequencing (WGS) and bioinformatics analysis

#### Sample preparation steps and genome sequencing

Isolates 1104–65 and 1104–75 were inoculated in Luria-Bertani broth and cultured at 37°C in a 200-rpm shaker until it reached a logarithmic phase. The broth was centrifuged at 10,000 rpm for 10 min at 4°C. After centrifugation, the supernatant was removed and rinsed 3–5 times with sterile water until the supernatant were clear. Samples were placed on dry ice for transportation immediately after sampling. All library preparation and sequencing were performed by the Novogene Bioinformatics Technology (Tianjin, China).

#### Analysis of whole genome sequencing data

Sequence reads were assembled using Unicycler 0.4.8 (64) and annotated using Prokka 1.14.5 (65). The predicted serotype and multi-locus sequence typing (MLST) types were identified using the Salmonella *in Silico* Typing Resource (SISTR 1.1.1) (66), and MLST 2.18.0 (67). The antibiotic-resistance genes and plasmid replicons were predicted using ResFinder 4.1 (68) and PlasmidFinder 2.1 (69), respectively, the default parameters were applied with minimum thresholds of sequence identity (>90%) and sequence coverage (>60%). Transposon and insertion sequence (IS) elements were scanned using the ISfinder database (70). Phylogenetic analysis was performed using Parsnp (71), the phylogenetic tree was visualized using Evolview online (72), and the SNPs among the core genomes of NTS were determined by using MEGA X (73). Close relatedness of isolates was defined as  <21 allele differences in cgMLST (74). The genetic environment was visualized by the EasyFig software (75) and Adobe Illustrator (AI).

### Conjugation experiments

Rifampicin-resistant *E. coli* C600 was used as the recipient strain and imipenem-resistant isolate 1104–75 was used as donor strain to determine the transferability of carbapenem-resistance phenotype. Transconjugant was selected on Luria–Bertani plates containing 100 µg/mL rifampicin plus 2 µg/mL imipenem, and the resistance phenotype was investigated by AST. PCR and sequencing were used to confirm whether the transconjugant carried the carbapenem resistance gene (NDM-F: ATGGAATTGCCCAATATTATGCAC, NDM-R: TCAGCGCAGCTTGTCGGC) and the related plasmid replicon (FII-F: CTGATCGTTTAAGGAATTTT, FII-R: CACACCATCCTGCACTTA).

## Data Availability

The nucleotide sequences of the genomes and plasmids of 1104–75 and 1104–65 have been uploaded to GenBank under the accession numbers CP110198-CP110200 and CP110201, respectively.

## SUPPLEMENTAL MATERIAL

Fig. S1 msphere.00480-23-s0001.pdf Phylogenetic analysis between 1104-65, 1104-75, ATCC14028, and other *S. enterica* serovar Typhimurium in this area.

Table S1 msphere.00480-23-s0002.pdf Information about other 67 strains of *S. enterica* serovar Typhimurium isolated in this area.

ASM does not own the copyrights to Supplemental Material that may be linked to, or accessed through, an article. The authors have granted ASM a non-exclusive, world-wide license to publish the Supplemental Material files. Please contact the corresponding author directly for reuse.
