# Supplementary material for: Molecular characterization of IncFII plasmid carrying blaNDM-5 in a Salmonella enterica serovar Typhimurium ST34 clinical isolate in China
Source: mSphere. 2023 Nov 1;8(6):e00480-23. doi: 10.1128/msphere.00480-23 (PMC10732066; doi:10.1128/msphere.00480-23)
Supplement: Fig. S1 — Phylogenetic analysis between 1104-65, 1104-75, ATCC14028, and other S. enterica serovar Typhimurium in this area. [file msphere.00480-23-s0001.pdf]

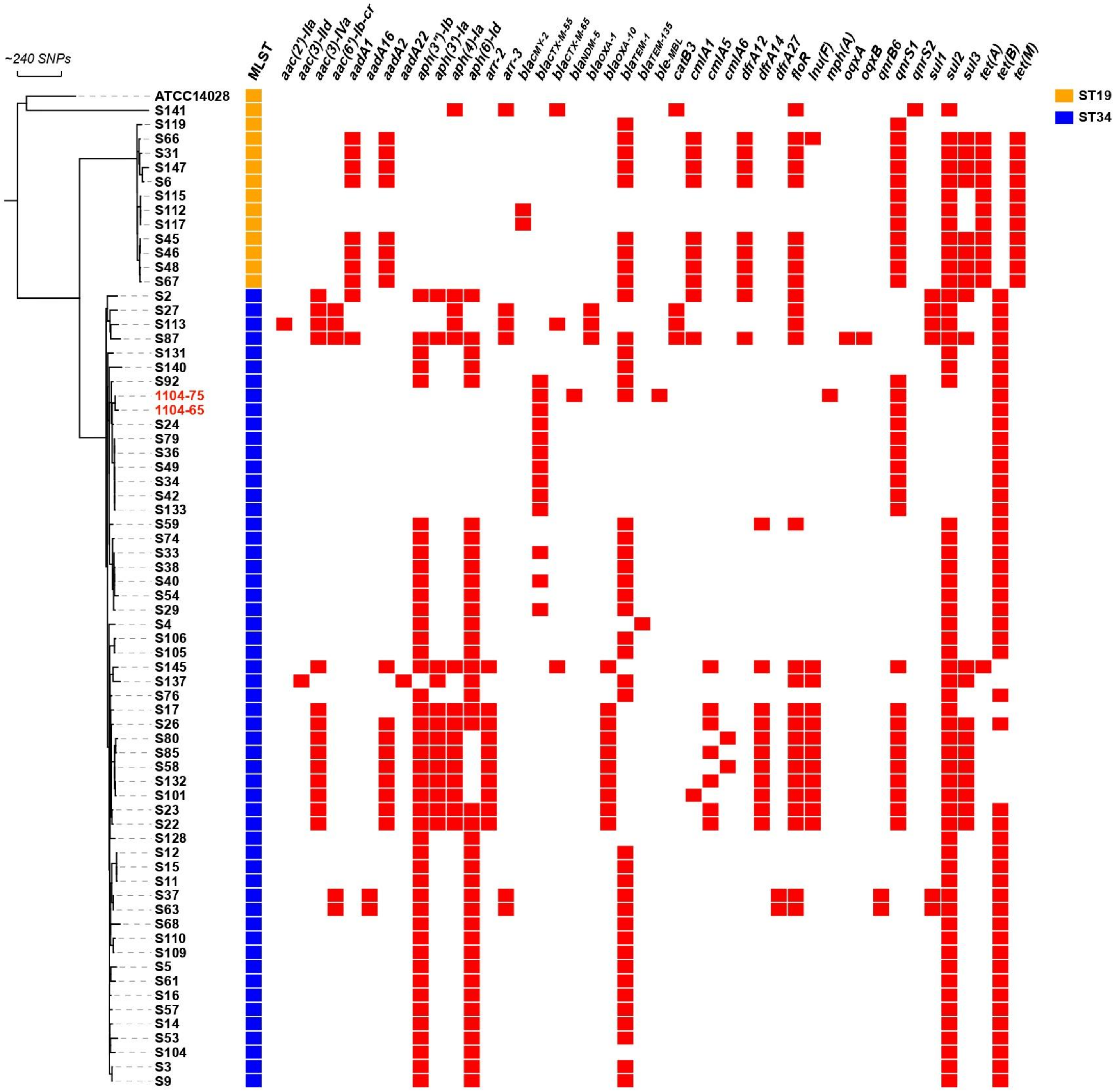

Supplementary Figure 1

Phylogenetic analysis between 1104-65, 1104-75, ATCC14028, and other *S. enterica* serovar Typhimurium in this area

The red font indicates the strain of this study, the yellow box indicates ST19, and the blue box indicates ST34. Red boxes represent the resistance genes for the different types of antibiotics, while the blank boxes indicate the resistance genes which were not detected.
