# Supplementary material for: Molecular characterization of IncFII plasmid carrying blaNDM-5 in a Salmonella enterica serovar Typhimurium ST34 clinical isolate in China
Source: mSphere. 2023 Nov 1;8(6):e00480-23. doi: 10.1128/msphere.00480-23 (PMC10732066; doi:10.1128/msphere.00480-23)
Supplement: Table S1 — Information about other 67 strains of S. enterica serovar Typhimurium isolated in this area. [file msphere.00480-23-s0002.pdf]

Supplementary Table 1 Information about other 67 strains of *S. enterica* serovar Typhimurium isolated in this area

Isolates	Serotype	ST	Isolation date	MIC (mg/L)						KB (mm)			
				SXT	FEP	CRO	CAZ	IPM	LVX	AZM	AMP	CIP	CHL
S2	Typhimurium	34	2020/5/23	>=320	<=0.12	<=0.25	0.5	<=0.25	1	22	6	26	6
S3	Typhimurium	34	2020/5/23	<=20	<=0.12	<=0.25	0.25	<=0.25	<=0.12	24	6	35	31
S4	Typhimurium	34	2020/5/23	<=20	<=0.12	<=0.25	0.25	<=0.25	<=0.12	24	6	34	32
S5	Typhimurium	34	2020/5/25	<=20	<=0.12	<=0.25	0.25	<=0.25	<=0.12	23	6	35	29
S6	Typhimurium	19	2020/5/25	<=20	<=0.12	<=0.25	1	<=0.25	<=0.12	24	6	36	31
S9	Typhimurium	34	2020/6/3	<=20	<=0.12	<=0.25	0.25	<=0.25	<=0.12	24	6	35	30
S11	Typhimurium	34	2020/6/5	<=20	<=0.12	<=0.25	<=0.25	<=0.25	<=0.12	24	6	35	29
S12	Typhimurium	34	2020/6/6	<=20	<=0.12	<=0.25	0.25	<=0.25	<=0.12	23	6	35	30
S14	Typhimurium	34	2020/6/11	<=20	<=0.12	<=0.25	0.25	<=0.25	<=0.12	22	6	34	30
S15	Typhimurium	34	2020/6/11	<=20	<=0.12	<=0.25	0.25	<=0.25	<=0.12	22	6	37	31
S16	Typhimurium	34	2020/6/15	<=20	<=0.12	<=0.25	0.25	<=0.25	<=0.12	22	6	34	32
S17	Typhimurium	34	2020/6/15	>=320	<=0.12	<=0.25	0.25	<=0.25	1	26	9	25	6
S22	Typhimurium	34	2020/6/19	<=20	<=0.12	<=0.25	<=0.12	<=0.25	0.5-	23	6	26	6
S23	Typhimurium	34	2020/6/20	>=320	<=0.12	<=0.25	0.25	<=0.25	1	24	6	24	6
S24	Typhimurium	34	2020/6/24	<=20	16	>=64	32	<=0.25	1	25	6	26	31
S26	Typhimurium	34	2020/6/27	>=320	<=0.12	<=0.25	0.25	<=0.25	1	25	6	25	6
S27	Typhimurium	34	2020/6/28	<=20	<=0.12	<=0.25	0.25	<=0.25	1	24	6	21	6
S29	Typhimurium	34	2020/7/2	<=20	16	>=64	>=64	<=0.25	<=0.12	26	6	36	30
S31	Typhimurium	19	2020/7/3	>=320	<=0.12	<=0.25	<=0.12	<=0.25	1	26	6	26	6
S33	Typhimurium	34	2020/7/2	<=20	16	>=64	>=64	<=0.25	<=0.12	25	6	37	32
S34	Typhimurium	34	2020/7/5	<=20	16	>=64	32	<=0.25	1	25	6	27	31
S36	Typhimurium	34	2020/7/6	<=20	16	>=64	32	<=0.25	1	27	6	28	31
S37	Typhimurium	34	2020/7/5	>=320	<=0.12	<=0.25	0.25	<=0.25	1	25	6	21	6

S38	Typhimurium	34	2020/7/6	<=20	<=0.12	<=0.25	<=0.12	<=0.25	<=0.12	24	6	36	32
S40	Typhimurium	34	2020/7/7	>=320	<=0.12	<=0.25	0.25	<=0.25	1	25	6	27	6
S42	Typhimurium	34	2020/7/8	<=20	>=32	>=64	>=64	<=0.25	1	26	6	25	29
S45	Typhimurium	19	2020/7/10	>=320	<=0.12	<=0.25	<=0.12	<=0.25	1	25	6	26	6
S46	Typhimurium	19	2020/7/10	>=320	<=0.12	<=0.25	0.25	<=0.25	1	25	6	25	6
S48	Typhimurium	19	2020/7/12	>=320	<=0.12	<=0.25	<=0.12	0.5	1	24	6	24	6
S49	Typhimurium	34	2020/7/11	<=20	8	>=64	32	<=0.25	1	25	6	25	28
S53	Typhimurium	34	2020/7/14	<=20	<=0.12	<=0.25	0.25	<=0.25	<=0.12	26	6	34	31
S54	Typhimurium	34	2020/7/15	<=20	<=0.12	<=0.25	0.25	<=0.25	<=0.12	24	6	37	30
S57	Typhimurium	34	2020/7/17	<=20	<=0.12	<=0.25	0.25	<=0.25	<=0.12	25	6	35	30
S58	Typhimurium	34	2020/7/17	>=320	<=0.12	<=0.25	0.25	<=0.25	1	26	6	25	6
S59	Typhimurium	34	2020/7/17	>=320	<=0.12	<=0.25	<=0.12	<=0.25	<=0.12	25	6	34	7
S61	Typhimurium	34	2020/7/18	<=20	<=0.12	<=0.25	0.25	<=0.25	<=0.12	25	6	36	30
S63	Typhimurium	34	2020/7/19	>=320	<=0.12	<=0.25	0.25	<=0.25	1	25	6	20	6
S66	Typhimurium	19	2020/7/20	>=320	<=0.12	<=0.25	<=0.12	<=0.25	1	24	6	25	6
S67	Typhimurium	19	2020/7/20	>=320	<=0.12	<=0.25	<=0.12	<=0.25	1	24	6	26	6
S68	Typhimurium	34	2020/7/22	<=20	<=0.12	<=0.25	0.25	<=0.25	<=0.12	25	6	34	33
S74	Typhimurium	34	2020/7/28	<=20	<=0.12	<=0.25	0.25	<=0.25	<=0.12	24	6	34	28
S76	Typhimurium	34	2020/7/30	<=20	<=0.12	<=0.25	0.25	<=0.25	<=0.12	24	6	25	29
S79	Typhimurium	34	2020/8/10	<=20	8	>=64	32	<=0.25	1	26	6	28	30
S80	Typhimurium	34	2020/8/10	>=320	<=0.12	<=0.25	0.5	<=0.25	1	24	6	30	6
S85	Typhimurium	34	2020/8/21	>=320	<=0.12	<=0.25	0.25	<=0.25	1	23	6	25	6
S87	Typhimurium	34	2020/8/25	>=320	<=0.12	<=0.25	0.25	<=0.25	4	24	6	16	6
S92	Typhimurium	34	2020/9/1	<=20	16	>=64	32	<=0.25	1	25	6	27	30
S101	Typhimurium	34	2020/9/9	>=320	<=0.12	<=0.25	0.5	<=0.25	1	24	6	25	6
S104	Typhimurium	34	2020/9/14	<=20	<=0.12	<=0.25	0.25	<=0.25	<=0.12	22	23	34	31

S105	Typhimurium	34	2020/9/14	<=20	<=0.12	<=0.25	0.5	<=0.25	<=0.12	23	6	34	30
S106	Typhimurium	34	2020/9/16	<=20	<=0.12	<=0.25	0.5	<=0.25	<=0.12	22	6	33	28
S109	Typhimurium	34	2020/9/19	>=320	2	<=0.25	4	<=0.25	1	22	6	35	32
S110	Typhimurium	34	2020/9/24	<=20	<=0.12	<=0.25	0.5	<=0.25	<=0.12	25	6	34	29
S112	Typhimurium	19	2020/9/26	<=20	<=0.12	32	32	<=0.25	1	25	6	25	30
S113	Typhimurium	34	2020/9/28	<=20	2	>=64	4	<=0.25	1	25	6	20	6
S115	Typhimurium	19	2020/9/29	<=20	<=0.12	<=0.25	0.25	<=0.25	1	26	26	24	31
S117	Typhimurium	19	2020/10/3	>=320	<=0.12	<=0.25	0.5	<=0.25	1	26	6	25	31
S119	Typhimurium	19	2020/10/6	<=20	<=0.12	<=0.25	0.25	<=0.25	1	27	6	25	28
S128	Typhimurium	34	2020/10/8	<=20	<=0.12	<=0.25	0.25	<=0.25	<=0.12	24	24	36	28
S131	Typhimurium	34	2020/10/26	<=20	<=0.12	<=0.25	<=0.12	<=0.25	1	24	6	26	29
S132	Typhimurium	34	2020/11/1	>=320	<=0.12	<=0.25	0.25	<=0.25	1	25	6	26	6
S133	Typhimurium	34	2020/11/5	<=20	8	>=64	32	<=0.25	1	25	6	27	30
S137	Typhimurium	34	2020/11/24	<=20	<=0.12	<=0.25	0.5	<=0.25	0.5	23	6	31	6
S140	Typhimurium	34	2020/12/12	<=20	<=0.12	<=0.25	0.25	<=0.25	0.5	26	6	30	30
S141	Typhimurium	19	2020/12/24	<=20	2	>=64	4	<=0.25	1	25	6	26	6
S145	Typhimurium	34	2021/2/14	>=320	16	>=64	4	<=0.25	1	25	6	24	6
S147	Typhimurium	19	2021/2/17	>=320	<=0.12	<=0.25	0.25	<=0.25	1	26	6	25	6
